# Routine Computed Tomography Versus Selective Imaging: An Audit of Negative Appendicectomy Rates in Two Hospitals

**DOI:** 10.7759/cureus.32389

**Published:** 2022-12-10

**Authors:** Tristan Symonds, Helen Buschel, John Avramovic, Pranavan Palamuthusingam

**Affiliations:** 1 Orthopaedics, Ipswich General Hospital, Ipswich, AUS; 2 General Surgery, Townsville University Hospital, Townsville, AUS

**Keywords:** imaging, computer tomography, ultrasonography, appendectomy, negative appendectomy rate, appendicitis

## Abstract

Introduction

There are a variety of conflicting recommendations in the literature for pre-operative imaging in acute appendicitis. There is debate over what the ideal imaging protocol is to lower the negative appendicectomy rate (NAR) without increasing missed appendicitis. The aim of this study is to compare the audited NAR between two groups with different imaging approaches: (i) mandatory pre-operative computed tomography (CT) imaging and (ii) selective imaging with CT, ultrasound (US), or no imaging prior to appendicectomy.

Materials and methods

A retrospective chart audit was conducted of 400 patients who underwent an appendicectomy at two hospitals with different approaches to pre-operative imaging (hospital A and hospital B). The primary outcome measure was histologically confirmed appendicitis. It was also documented whether there was radiological (CT or US) evidence of appendicitis.

Results

At hospital A, all 200 patients underwent CT imaging prior to appendicectomy. The total histologically confirmed NAR for this group was 9.5% (19/200). At hospital B, 97 (48.5%) patients underwent CT, 41 (25.5%) underwent US, 10 (5%) had both US and CT, and 52 (26%) had no imaging. The total NAR was 11.5% (23/200).

Conclusion

There was no statistically significant difference (p=0.62) in audited NARs when comparing clinician-guided selective imaging versus routine CT imaging for all patients undergoing appendicectomy.

## Introduction

The reported negative appendicectomy rate (NAR) for acute appendicitis is variable and ranges from 10-40% [1-3]. A NAR of 10% is generally considered acceptable in practice, with an aim to reduce the number of missed appendicitis cases and subsequent complications (including perforation). However, a negative appendicectomy is associated with unnecessarily longer hospital admission, morbidity, cost, and use of health system resources [4,5]. To reduce the NAR, numerous authors advocate for the use of ultrasound (US) and/or computed tomography (CT). However, there is debate over the ideal imaging protocol for lowering the NAR without increasing the rate of missed appendicitis.

Several authors have reported a reduction in NAR with the introduction of CT imaging [6-9]. Given that pre-operative CT scanning is becoming more accessible and that the risk associated with modern CT radiation has declined [10], many propose routine CT scan to reduce NARs [4,11].

Alternatively, other studies endorse US as the first-line imaging for patients with suspected appendicitis [3]. The 2010 guidelines released by the Dutch College of Surgeons recommended that prior to appendicectomy all patients should undergo US imaging, followed by CT if the US is negative or inconclusive [12]. 

Various other recommendations have been proposed in the literature, including CT or US only for diagnostic uncertainty [13] and routine CT for male patients with selective imaging for female patients [2,14]. Other authors argue that clinical assessment unaided by CT reliably identifies patients requiring operative management and that pre-operative imaging causes avoidable delay [15]. Finally, the use of diagnostic laparoscopy for patients with negative imaging findings and an unclear clinical picture has been safely described without increasing the risk of complications [16,17].

This audit examines the NAR associated with different imaging protocols at two hospitals located in the same regional town. At hospital A, routine pre-operative CT was implemented following an internal audit that found the NAR was 17% with clinical diagnosis by five specialist general surgeons versus 1% with CT. This finding is comparable to reports in the literature of improved NAR with the use of CT [6,9]. On the other hand, hospital B employs a selective imaging approach where patients may undergo no imaging, US and/or CT based on clinical assessment prior to appendicectomy. The aim of this audit is to compare the NAR with both imaging protocols.

## Materials and methods

A retrospective chart review was conducted of 400 consecutive patients aged 18 and over, who underwent appendicectomy at two regional hospitals, Mater Townsville and Townsville University Hospital, during 2017-2018. Both hospitals are located in Townsville in Queensland, Australia; however, there were significant differences between the two institutions. Approval for the study was given by Townsville University Hospital Human Research Ethics Committee (approval number: LNR/QTHS/60983).

Mater Townsville (Hospital A) is a private hospital with easy access to imaging and consultant surgeon management of patients. Here, all patients undergo CT imaging reported by a consultant radiologist prior to appendicectomy. All histology was performed by a consultant pathologist. Townsville University Hospital (Hospital B), on the other hand, is a public hospital with higher demands for imaging and longer wait times. Depending on clinical assessment, patients may undergo no imaging, US, CT, or both prior to appendicectomy. Patients are initially managed by surgical registrars, at variable stages in their training, under the supervision of consultant surgeons. Imaging is primarily reported by a training radiology registrar and later reviewed by a consultant radiologist. Some consultant surgeons and radiologists work at hospitals A and B. All histology was performed by a consultant pathologist.

Data collected included patient demographics (age and gender), imaging performed (CT, US, or both), whether imaging was positive or negative for appendicitis, and histologically confirmed or disproved appendicitis. All CT scans were performed with contrast.

Inclusion and exclusion criteria

All adult patients 18 years and over who underwent an emergency appendicectomy were included in the study. Exclusion criteria were: pregnant patients, incidental appendicectomy during an unrelated procedure, and patients undergoing elective interval appendicectomy.

Statistical analysis

The primary outcome measure was NAR based on histological diagnoses. The secondary outcome was radiological diagnosis by US and/or CT. Statistical significance for the analysis of dichotomous variables was determined using Pearson chi-square tests with a 0.05% significance.

## Results

Hospital A

At hospital A, 200 appendicectomies were audited. The results are demonstrated in Table 1. There were 102 (51%) male patients and the mean age was 46 years (range 18-80). All 200 patients had a CT prior to appendicectomy. Of these patients, 182 (91%) had CT-confirmed acute appendicitis and 18 (9%) were radiologically negative for appendicitis. The total histologically-confirmed NAR for this hospital was 9.5% (19 patients). 

**Table 1:** Hospital A: radiological and pathological findings (histologically confirmed appendicitis) † represents negative appendicectomy rate (NAR)

Imaging (CT) finding	Pathology positive	Pathology negative	Total
CT positive	178	4	182
CT negative	3	15	18
Total	181	19 (9.5%)^†^	200

In the patient population with CT-diagnosed appendicitis, there was a NAR of 2.2% (four out of 182). Three out of 18 patients who had CT scans negative for appendicitis had histologically confirmed appendicitis, a rate of 1.5% radiologically missed appendicitis.

Hospital B

At hospital B, 200 appendicectomies were performed. The results demonstrating the imaging prior to appendectomy are shown in Table 2. There were 91 male patients (45.5%) and the mean age was 39 years (range 18-79). Fifty-two patients (26%) were assessed clinically to have appendicitis and proceeded to operation without imaging. Forty-one patients (25.5%) underwent US only, 97 patients (48.5%) had a CT scan only, and 10 patients (5%) underwent both US and CT scans prior to appendicectomy. Table 3 demonstrates the results based on the imaging and pathological findings.

**Table 2:** Hospital B: imaging prior to appendicectomy

Imaging finding	No Imaging	US only	CT only	US and CT
Radiology positive	-	24	90	10
Radiology negative	-	17	7	0
Total	52	41	97	10

**Table 3:** Hospital B: imaging and pathological findings (histologically confirmed appendicitis) † represents negative appendicectomy rate (NAR); ‡ eight CT positive and two US positive

Imaging	Pathology positive	Pathology negative	Total
No imaging	42	10 (19.2%^†^)	52
US scan only			
Positive US	23	1	24
Negative US	10	7	17
Total	33	8 (19.5%^†^)	41
CT scan only			
Positive CT	88	2	90
Negative CT	4	3	7
Total	92	5 (5.2%^†^)	97
Both US and CT scan			
Positive imaging	10^‡^	0	10
Negative imaging	0	0	0
Total	10	0 (0%^†^)	10
Total	177	23	200

No-imaging Group

Ten out of the 52 patients who underwent no imaging had a normal appendix histologically, a NAR of 19.2%. 

US-only Group

Of the 41 patients who underwent US scans only, 24 (58.3%) were radiologically positive for appendicitis and 17 (41.4%) were radiologically negative. The NAR for patients examined by ultrasound alone was 19.5% (eight out of 41 patients). 

CT-only Group

In the CT-only group, 90 (92.8%) had CT-proven appendicitis. The NAR for patients who underwent a CT was 5.2% (five out of 97). In patients who had a CT positive for appendicitis, the NAR was 2.2% (two out of 90 patients), the same as hospital A. Four out of seven patients had CT missed appendicitis (4%).

Both-CT-and-US Group

Of the 10 patients who underwent both US and CT imaging at hospital B, all 10 patients were radiologically positive for appendicitis. Eight out of 10 had a positive CT scan and two out of 10 had a positive US scan. The NAR for this group was 0%.

The total NAR for hospital B was 11.5% (23 out of 200 patients).

Overall NAR

The NAR at hospitals A and B are shown in Table 4. The total NAR for both hospitals is comparable, with routine use of CT imaging at hospital A having a NAR of 9.5% (95%CI 5.4-13.5%) and selective imaging at hospital B having a NAR of 11.5% (95%CI 7.1-15.9). There was no statistically significant difference between routine CT imaging and selective imaging for acute appendicitis (p = 0.62). 

**Table 4:** Overall NAR in hospital A and hospital B † represents negative appendicectomy rate (NAR)

Pathology findings	Hospital A	Hospital B	Total
Pathology Positive	181	177	358
Pathology Negative	19 (9.5%^†^)	23 (11.5%^†^)	42
Total	200	200	400

## Discussion

This study compared the audited NAR at two hospitals with different imaging policies prior to appendicectomy. Mandatory pre-operative CT at hospital A and selective imaging at hospital B were associated with a NAR of 9.5% and 11.5%, respectively. There was no statistically significant difference in NAR (p=0.62).

There are multiple reports in the literature of improved NAR with CT [6,9]. Rao et al. presented one of the earliest studies in 1999 showing a decrease in NAR from 20% to 7% with the use of CT [8]. A meta-analysis of 28 studies in 2011 reported an average NAR of 16.7% with clinical evaluation alone versus 8.7% with the use of CT [7]. This is comparable to the 2012-2014 internal audit at hospital A that found a NAR of 17% with clinical diagnosis and 1% with CT.

At hospital B, the NAR in the clinical assessment group was 19.2% versus 5.4% in patients who underwent CT. An internal audit performed at hospital B in 2012 found the overall NAR was 28% when CT was used in 38% of cases, compared with 11.5% in this current audit with CT used in 48.5% of cases. These findings and our results further support the use of pre-operative CT imaging to reduce NAR, but not necessarily routine use of CT.

There are limited studies investigating NAR with mandatory CT versus selective imaging. A large 18-year retrospective study compared the NAR where CT was used in 1% of cases (1990-1994) with CT in 97.5% (2003-2007). The NAR improved from 23% to 1.7% with the frequent use of CT [18]. In 2007, Lee et al. performed a randomised controlled trial comparing selective and mandatory CT in the evaluation of right lower quadrant pain. For the 82 patients who underwent appendicectomy, there was a NAR of 13.9% in the selective CT group versus 2.6% in the mandatory group. Although this was not a statistically significant finding, the result showed a trend toward improvement in NAR with pre-operative CT [11]. These findings differ from the results of the present study, where there was no significant difference between audited NAR in routine CT versus selective imaging.

If clinicians had followed the imaging result alone, the NAR would be significantly lower with mandatory CT at hospital A (2.2%) compared to selective imaging at hospital B (9.5%). However, this would come at the expense of patients who had a falsely negative CT (i.e. radiologically missed appendicitis). Between the two hospitals, 25 patients proceeded to appendicectomy despite having a CT negative for appendicitis. Of these, three out of 18 at hospital A and four out of seven at hospital B with a negative CT had histologically confirmed appendicitis. Although clinical input was associated with a higher NAR, this was necessary to avoid missed appendicitis and the associated morbidity for these seven patients (1.75%).

A similar number of patients had a falsely negative CT at both hospitals; however, significantly more patients with a negative CT underwent appendicectomy at hospital A (18 versus seven). It is beyond the scope of this audit to assess why patients with a negative CT scan underwent an appendicectomy. However, we hypothesise that contributing factors may include persistent symptoms, repeat presentations with right iliac fossa pain, and high clinical suspicion resulting in diagnostic laparoscopy and appendicectomy.

At hospital B, 20.5% of patients underwent US only prior to appendicectomy, 85.4% of these patients were female. There are mixed reports in the literature on the diagnostic use of US prior to appendicectomy. A prospective study of 139 patients in Spain published in 2019 reported a NAR of 0% with routine US prior to appendicectomy [19]. Conversely, in 2016, Sauvain et al. retrospectively analysed 2559 Swiss patients and found CT significantly reduced NAR (9.3% to 5%), but there was no significant difference with the use of US [20].

In the present study, the use of US at hospital B resulted in a NAR of 19.5%, which was similar to the NAR of 19.2% for patients who had no imaging prior to appendicectomy. An inconclusive US held little value in determining whether a patient had appendicitis or not, with a negative predictive value of 41.18%. However, a positive US was useful with a positive predictive value of 95.83%.

Interestingly, the NAR for patients who underwent both US and CT imaging was 0%. However, within this group, 80% of CT scans and only 20% of US scans were positive for appendicitis. It is not possible to make further comments on this group due to the small sample size of 10 patients.

This study is limited by the fact that it is a retrospective audit. Therefore, other factors were not included in data collection including associated cost, clinical reasoning for the choice of selective imaging, reasons for diagnostic laparoscopy, presence or absence of intraabdominal abscess formation, or conversion to open procedure. US reports are often variable and subjective; a positive or negative radiographic finding can depend on the experience of the sonographer and this was not examined. Histology reports can be subjective; however, the reports in this study were binary for histology-confirmed or excluded appendicitis. Patients who underwent a CT and did not progress to appendicectomy were also not included. 

## Conclusions

In this retrospective audit, there was no difference in NAR between routine CT imaging and selective imaging (including no imaging, US, and/or CT). Routine CT imaging was not associated with a reduction in NAR when overriding clinical judgment is used. These results also highlight the importance of clinical judgment in preventing missed appendicitis due to false negative imaging.
